# Clinical implementation of a knowledge based planning tool for prostate VMAT

**DOI:** 10.1186/s13014-017-0814-z

**Published:** 2017-05-08

**Authors:** Richard Powis, Andrew Bird, Matthew Brennan, Susan Hinks, Hannah Newman, Katie Reed, John Sage, Gareth Webster

**Affiliations:** 1 Worcestershire Oncology Centre, Worcestershire Acute Hospitals NHS Trust, Worcester, UK; 2 Centre for Technology Enabled Health Care, Coventry University, Coventry, UK

**Keywords:** Knowledge-based planning (KBP), Plan optimisation, Organ at risk dose sparing, Scripting

## Abstract

**Background:**

A knowledge based planning tool has been developed and implemented for prostate VMAT radiotherapy plans providing a target average rectum dose value based on previously achievable values for similar rectum/PTV overlap. The purpose of this planning tool is to highlight sub-optimal clinical plans and to improve plan quality and consistency.

**Methods:**

A historical cohort of 97 VMAT prostate plans was interrogated using a RayStation script and used to develop a local model for predicting optimum average rectum dose based on individual anatomy. A preliminary validation study was performed whereby historical plans identified as “optimal” and “sub-optimal” by the local model were replanned in a blinded study by four experienced planners and compared to the original clinical plan to assess whether any improvement in rectum dose was observed. The predictive model was then incorporated into a RayStation script and used as part of the clinical planning process. Planners were asked to use the script during planning to provide a patient specific prediction for optimum average rectum dose and to optimise the plan accordingly.

**Results:**

Plans identified as “sub-optimal” in the validation study observed a statistically significant improvement in average rectum dose compared to the clinical plan when replanned whereas plans that were identified as “optimal” observed no improvement when replanned. This provided confidence that the local model can identify plans that were suboptimal in terms of rectal sparing. Clinical implementation of the knowledge based planning tool reduced the population-averaged mean rectum dose by 5.6Gy. There was a small but statistically significant increase in total MU and femoral head dose and a reduction in conformity index. These did not affect the clinical acceptability of the plans and no significant changes to other plan quality metrics were observed.

**Conclusions:**

The knowledge-based planning tool has enabled substantial reductions in population-averaged mean rectum dose for prostate VMAT patients. This suggests plans are improved when planners receive quantitative feedback on plan quality against historical data.

## Background

Volume Modulated Arc Therapy (VMAT) is a popular method of radiotherapy treatment delivery enabling high doses of radiation to be shaped to the treatment plan target volume (PTV) compared to conventional conformal radiotherapy techniques. VMAT plans are inherently complex and are typically produced in commercially available treatment planning systems via a user-informed inverse-planning process.

An optimum solution would deliver the full prescription dose to the planning target volume whilst delivering the lowest possible dose to surrounding organ at risk (OAR) structures. However, the variation in quality of a VMAT plan is ultimately influenced by numerous factors including the number of target structures and nearby organs-at-risk, the patient anatomy, planner experience and skill and the time available to produce the plan.

The RayStation clinical database at Worcester contains a record of the plan solutions produced locally for all historical patients. For a given patient encountered in the clinic it is likely that there already exists a patient with a similar geometric distribution of target and OAR structures. Furthermore, clinically acceptable plans exist for these patients creating a knowledge base which could be harnessed to inform users of achievable plan quality during future plan optimisations and to potentially drive improvements in plan quality over time.

A variety of different knowledge based planning tools are described in the literature with varying scope and complexity [1–8]. Moore et al. and Wu et al. independently reported that the level of achievable OAR dose sparing is related to the geometric arrangement of the OAR relative to the PTV [1, 2] and specifically the extent of overlap between the OAR and PTV. Moore et al. [1] assessed the relationship between mean OAR dose (D_mean_) and the volume of OAR overlapping a PTV (V_ovr_) for a knowledge base of historical patients. They constructed the following mathematical model for predicting D_mean_ based upon the fractional OAR-PTV overlap (V_ovr_/V_OAR_),

1 $$ \frac{D_{mean}}{D_{Px}}= A+ B\left(1- exp\left( C\frac{V_{ovr}}{V_{OAR}}\right)\right) $$

where A, B and C are coefficients selected to represent the optimal plans (in terms of OAR dose) in the knowledge base. The mathematical model was incorporated into a script in the planning system which presented the planner with a predicted value D_mean_ for the chosen patient. The planner was then expected to optimise the plan in order to achieve an OAR dose less than or equal to D_mean_. This approach was found to lower the risk of plans being produced with sub-optimal OAR average dose and to reduce variation between planners.

Wu et al. investigated OAR and PTV overlap via the more sophisticated concept of the overlap volume histogram (OVH) [2]. The OVH was then used to identify patients in a historical database with similar geometric relationships between the PTV and surrounding OAR whose dose volume histogram (DVH) were used to guide the plan optimisation process. Appenzoller et al. [3] built upon the work of Moore et al. to develop a mathematical model for predicting the achievable DVH for an OAR using the correlation between expected dose at a point and the vicinity of that point to the PTV. The predicted DVH curves were successfully used to guide the plan optimisation process and to identify outlier sub-optimal plans. Schriebmann et al. [4] proposed a “feature-selection” search method that identified cases in a historical database of VMAT plans with similar anatomy to the current patient. Once identified, the plan configuration and DVH statistics of the similar plan were utilised as a starting point for the new plan to aid and speed up the plan optimisation process. Nwankwo et al. [5] developed a knowledge based planning algorithm that predicts the 3D dose distribution of an OAR based upon its proximity to the OAR.

This paper describes the implementation of the method developed by Moore et al. at the Worcestershire Oncology Centre using the RayStation treatment planning system. It establishes a locally relevant predictive model, validates its use in removing sub-optimal plans and describes the impact of a controlled implementation. The Moore et al. model was chosen from the aforementioned knowledge based planning solutions in the literature due to the demonstrated potential for significant clinical benefit whilst being relatively simple to implement locally within the RayStation scripting interface.

## Methods

This work has focussed upon external beam radiotherapy of prostate patients at the Worcestershire Oncology Centre (WOC) which is delivered according to the CHHiP trial (CRUK/06/016) protocol. Patients are planned with three concentric target volumes PTV3, PTV2 and PTV1 prescribed 74Gy, 71Gy and 59.2Gy in 37 fractions respectively. Patients are separated into two categories depending upon whether they present a high or low risk of seminal-vesicle involvement. For high risk (HR) patients PTV3 is equal to the prostate outline plus a 5 mm isotropic margin (0 mm posteriorly), PTV2 is the prostate and seminal vesicles combined plus a 10 mm isotropic margin (5 mm posteriorly) and PTV1 is the prostate and seminal vesicles combined plus a 10 mm isotropic margin. For low risk (LR) patients the PTV structures are grown in the same manner as described for HR however PTV2 is grown from the prostate outline only.

### Method A: forming a local knowledge base for prostate planning

A script (henceforth referred to as the data-mining script) was produced within the RayStation scripting interface to interrogate a historic cohort of 97 prostate patients planned and treated between February 2015 and February 2016 at WOC. All patients were treated with single arc 6MV photon VMAT plans, received no nodal irradiation and had no artificial hip implants. The patients were distributed evenly between the disease-risk sub-cohorts with 49 patients designated as HR and 48 as LR.

For each patient the script extracted or calculated a variety of data for analysis including: ROI volumes, PTV-OAR overlap volumes, OAR mean doses (including average rectum dose), DVH data for PTV and OAR structures and plan quality/complexity metrics such as conformity index and total plan MU. In this work the conformity index (CI) is defined as the ratio between the volume of a target covered by a user specified isodose and the total volume covered by the isodose.

Using the data extracted by the script, the ratio of the average rectum dose (*D*_*mean*_^*rectum*^) to the primary prescription dose (*D*_*Px*_) of 74Gy to PTV3, and the ratio of the volume of rectum overlapping PTV1 (V_ovr_) to the overall volume of the rectum (V_rec_) was calculated for each patient. To assess whether the trend between average rectum dose and geometric OAR-PTV overlap reported by Moore et al. was evident in the historic cohort *D*_*mean*_^*rectum*^/*D*_*Px*_ was plotted against V_ovr_/V_rec_. The coefficients in Equation 1 were adjusted to provide an approximate fit the Moore et al. mathematical model to the local plot of *D*_*mean*_^*rectum*^/*D*_*Px*_ versus V_ovr_/V_rec_. This was done twice: firstly so that the mathematical model fit along the lower bound of the local data representing the “optimal average rectum dose” (OARD) achieved in the historic cohort and secondly so that the mathematical model fit through the middle of the historic cohort representing the “median average rectum dose” (MARD).

### Method B: validation of local model

If the OARD is a valid metric for identifying plans with “sub-optimal” rectal sparing (i.e. *D*_*mean*_^*rectum*^ > > OARD) it is hypothesised that re-planning those patients would yield an improvement in rectal sparing. Similarly, re-planning those patients with “optimal” original clinical plan (i.e. *D*_*mean*_^*rectum*^ ≈ OARD) would yield little or no change in rectal sparing.

Moore et al. defined the relative model excess, δ, to quantify the difference in achieve mean rectal dose compared to the predicted value (D_pred_) as follows,

2 $$ \delta =\frac{D_{mean} - {D}_{Pred}}{D_{pred}} $$

This metric was chosen as it is insensitive to absolute values in dose or overlap volume allowing comparison of plans across different sites and prescriptions [1]. A planning study was devised to test whether the OARD and MARD models could be used to assess if a plan was “optimal” (compared to the historical knowledge base) in terms of rectal sparing utilising the metric δ.

For each patient in the historic cohort δ was calculated using Equation 2 where D_pred_ was calculated using the local OARD model introduced in Method A: Forming a Local Knowledge Base for Prostate Planning. Ten patients were selected from the cohort; five patients with a high δ-value (where *D*_*mean*_^*rectum*^ > > OARD and implying that the original clinical plans were “sub-optimal” in terms of rectum dose) and five patients with δ ≈ 0 (*D*_*mean*_^*rectum*^ ≈ OARD) implying that the original clinical plans were close to the best achieved in the historical cohort). Prior to the study the ten plans were reviewed by an independent experienced planner to check that there were no mitigating circumstances explaining why each plan might exhibit a high rectum dose.

The patients were anonymised and four experienced planners were each asked to produce a new plan for each patient. The planners were provided with additional planning goals to ensure that the new plans maintained similar levels of PTV coverage and non-rectum OAR sparing compared to the original clinical plans. These additional goals included D99 targets for PTV1, PTV2, and PTV3, mean dose and D10 targets for the bladder and low dose conformity indices. Additionally, planners were asked to achieve rectal sparing where they felt it was achievable but were not informed as to which plans were predicted to have scope for rectal dose reduction.

The relative model excess, δ was calculated for each replan and the average δ across the four planners was calculated for each patient and compared to δ for the original clinical plan to assess if the plan average rectal dose had improved.

### Method C: implementation of local knowledge based planning tool

A RayStation script, henceforth referred to as the knowledge based planning (KBP) script, was developed for clinical implementation utilising the mathematical models for predicting OARD and MARD established in Method A: Forming a Local Knowledge Base for Prostate Planning. Upon execution the KBP script performs the following tasks:

- Determines the volume of the rectum ROI that overlaps the PTV1 target and calculates V_ovr_/V_rec_.
- Calculates the corresponding MARD and OARD using the mathematical models established in Method A: Forming a Local Knowledge Base for Prostate Planning.
- Displays the prescription dose, fractionation, V_ovr_/V_rec_, current plan average rectum dose, MARD and OARD in a graphical user interface.

Once the user has acknowledged the results graphical user interface (GUI) a message box is created advising the user that the current average rectum dose is either not-acceptable, acceptable or “optimal” depending upon whether *D*_*mean*_^*rectum*^ >MARD, MARD> *D*_*mean*_^*rectum*^ >OARD or *D*_*mean*_^*rectum*^ <= OARD respectively . Initial preliminary testing suggested that using the KBP script could lead to greater rectum sparing at the cost of an increase in plan MU, a slight reduction in PTV1 coverage (but still within the clinical goal) and a reduction in low dose conformity with an associated increase in femoral head dose (but again still within the clinical goal). Prior to implementing the KBP script clinically the benefits and potential costs were raised with local clinicians. The clinicians were willing to accept the aforementioned costs in order to obtain reductions in average rectum dose.

The KBP script was implemented clinically, with planners asked to ensure that the current plan average rectum dose was at least lower than the MARD and preferably equal to or lower than the OARD presented in the KBP script GUI. The data extracted from the data-mining script (see Method A: Forming a Local Knowledge Base for Prostate Planning) were used to determine values for PTV3 coverage, low dose conformity index and total plan MU that were routinely achieved in the historical cohort and were desirable in all future plans. As a precaution, these values were added as additional clinical goals to be met during the planning process in order to reduce the risk of the KBP script generating unforeseen changes in practice or plan quality.

## Results

### Results A: forming a local knowledge base for prostate planning

**Fig. 1:**
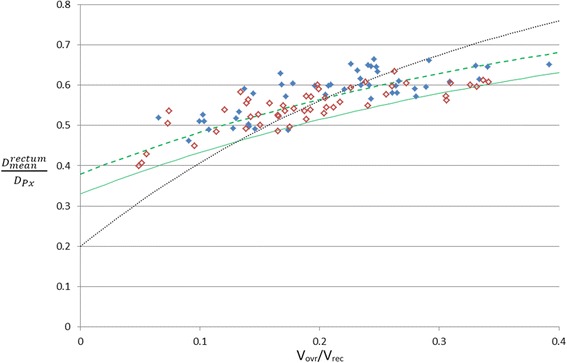
Average dose to the rectum normalised to the prescription dose (*D*_*mean*_^*rectum*^/*D*_*Px*_) plotted against the volume of the rectum overlapping PTV1 expressed as a fraction of total rectum volume (V_ovr_/V_rec_). Data is plotted for high risk (*solid*) and low risk (*open*) patients. The *dashed* and *solid curves* represent the local MARD and OARD models respectively whereas the *dotted curve* represents the model of Moore et al. [1]

Figure 1 displays (*D*_*mean*_^*rectum*^/*D*_*Px*_) plotted against (V_ovr_/V_rec_) for the WOC prostate historical cohort. The data presents a clear trend with average rectum dose increasing as the fractional overlap of the rectum and PTV1 increases which is consistent with the trend observed by Moore et al. The data displays no significant variation between the HR (solid data points) and LR (hollow data points) patient cohorts and throughout the remainder of this report the stratification according to disease risk is ignored (i.e. the data is treated as a single cohort).

The dotted curve is the mathematical fit reported by Moore et al. The solid curve represents the optimal (OARD) fit to the entire historical cohort described by equation 1 using coefficients A = 0.33, B = 0.5 and C = −2.3. The dashed curve represents the median (MARD) fit to the entire historical cohort described by equation 1 using coefficients A = 0.38, B = 0.5 and C = −2.3.

### Results B: preliminary testing of local model

The average change in δ was −0.08 (range −0.12 to −0.01) for the high-δ_clin_ cohort and −0.02 (range −0.06 to 0.00) for the low-δ_clin_ cohort. Applying a single-tailed paired *t*-test, the high-δ_clin_ cohort exhibits a statistically significant reduction in δ between the original clinical plans and the average of the replans (*p* = 0.009). This indicates that when re-planned these patients observed an overall reduction in mean rectum dose relative to the original clinical plan so that the planned average dose was closer to the OARD prediction. In contrast, the low-δ_clin_ cohort do not exhibit a statistically significant change in δ (*p* = 0.201). This suggests that when re-planned these patients observed only a very small change in mean rectum dose compared to the original clinical plan.

Whilst the preliminary study only contained five patients in each cohort (limiting its statistical power) overall the results support the hypothesis that the OARD can be used to identify plans which are “sub-optimal” in terms of average rectum dose. Despite the limited statistical power of this test, it provided sufficient confidence to proceed with the clinical implementation described in method C. The dose to the femoral heads was not actively controlled during the study however there was no systematic increase in femoral head D50 observed when comparing the replans to the original clinical plans.

### Results C: implementation of local knowledge based planning tool

**Fig. 2:**
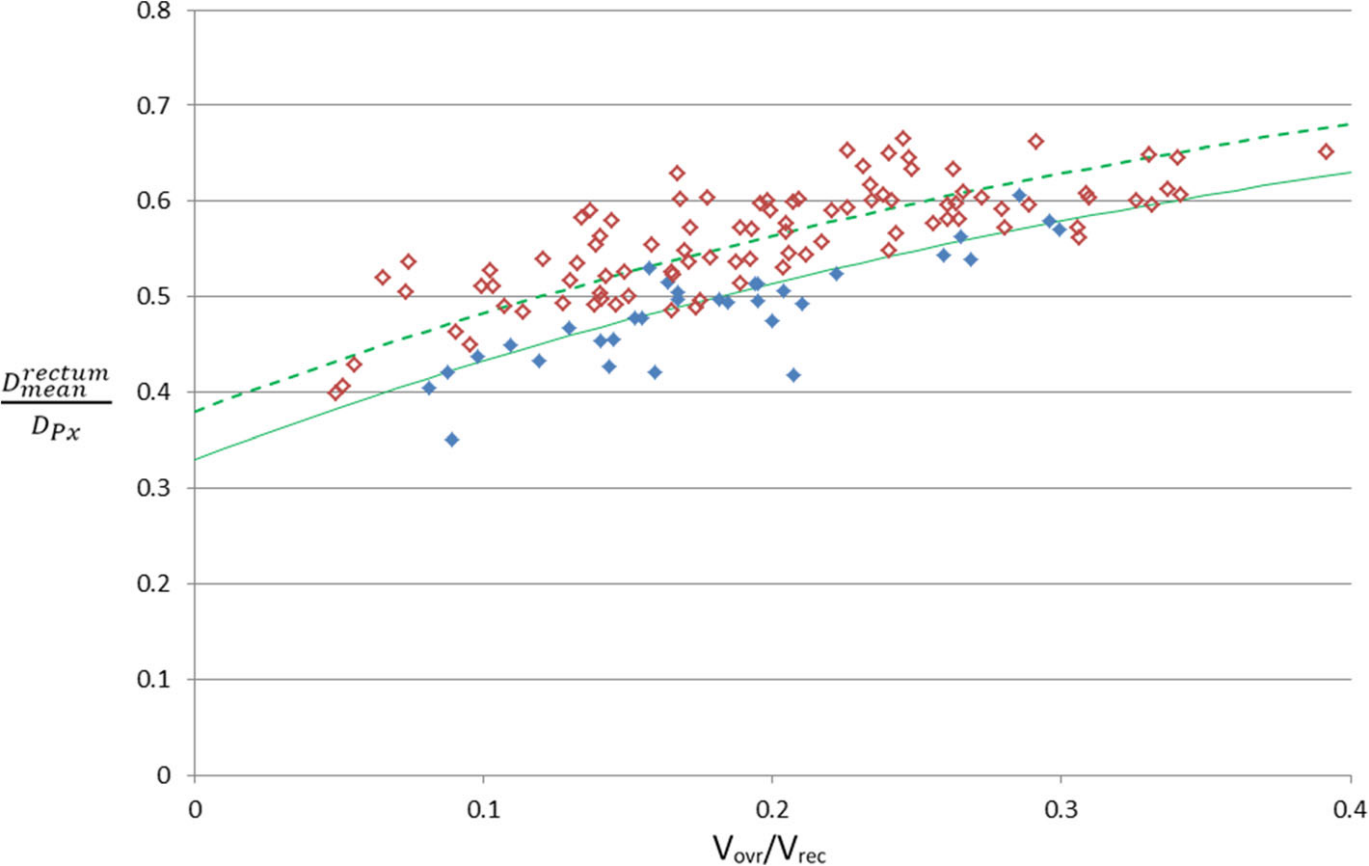
Average dose to the rectum normalised to the prescription dose (*D*_*mean*_^*rectum*^/*D*_*Px*_) plotted against the volume of the rectum overlapping PTV1 expressed as a fraction of total rectum volume (V_ovr_/V_rec_). Data is plotted for 33 patients planned post KBP script implementation (*solid*) and for the historical cohort (*open*). The *dashed* and *solid curves* represent the local MARD and OARD models respectively

Figure 2 displays *D*_*mean*_^*rectum*^/*D*_*Px*_ plotted against V_ovr_/V_rec_ for the historic patient cohort planned pre-KBP (open diamonds) and the first 33 patients post-KBP script implementation (filled-diamonds). All 33 plans produced post-script implementation met all clinical goals and were approved as clinically acceptable following the routine local plan checking and approval processes. The solid and dashed green lines in Fig. 2 represent the predicted OARD and MARD respectively for the historical cohort as introduced in Method A: Forming a Local Knowledge Base for Prostate Planning.

For the historical cohort (pre-KBP script implementation) the patients are distributed evenly around the MARD curve and the mean δ was 0.11 ± 0.08. Post-implementation of the KBP script the average rectum dose is reduced substantially so that patients are distributed around the OARD curve and every plan exhibits *D*_*mean*_^*rectum*^ < MARD. Post-script implementation the mean δ was −0.03 ± 0.06.

Table 1 displays mean values for a variety of plan statistics for the patient plans produced pre- and post-KBP script implementation, and *p*-values (calculated using a two-sample, two-tailed *t*-test assuming unequal variances) indicating whether the difference in the distribution of plan statistics pre- and post- script implementation are statistically significant (assuming a significance level of *p* < 0.05).

**Table 1:** Population-averaged plan statistics (standard deviation in parentheses) for the patients planned pre- and post- KBP script implementation

Metric	Pre	Post	*P*-value
PTV3 D99 (Gy)	72.4 (0.5)	72.4 (0.4)	0.748
PTV3 D1 (Gy)	75.1 (0.6)	75.1 (0.4)	0.904
PTV2 D99 (Gy)	67.6 (0.3)	67.7 (0.3)	0.471
PTV1 D99 (Gy)	59.3 (2.9)	59.7 (2.0)	0.351
Total MU	428 (70)	463 (73)	0.018
Av Bladder dose (Gy)	23.2 (8.8)	22.3 (7.8)	0.580
Av Rectum dose (Gy)	41.6 (4.2)	36.0 (4.1)	<0.001
Av bowel dose (Gy)	23.7 (19.9)	14.1 (14.8)	0.289
LFemH D50 (Gy)	21.7 (7.1)	26.3 (5.6)	<0.001
RFemH D50 (Gy)	21.6 (7.3)	27.2 (7.1)	<0.001
CI (56.2Gy to PTV1)	0.69 (0.06)	0.65 (0.04)	<0.001
CI (40.0Gy to PTV1)	0.37 (0.04)	0.34 (0.03)	<0.001
δ	0.11 (0.08)	−0.02 (0.06)	<0.001
Rectum V30/V_rec_	0.71 (0.07)	0.54 (0.10)	<0.001
Rectum V40/V_rec_	0.54 (0.11)	0.38 (0.01)	<0.001
Rectum V50/V_rec_	0.37 (0.11)	0.28 (0.08)	<0.001
Rectum V60/V_rec_	0.21 (0.10)	0.18 (0.06)	0.110
Rectum V65/V_rec_	0.13 (0.08)	0.13 (0.05)	0.787
Rectum V70/V_rec_	0.07 (0.04)	0.07 (0.03)	0.457

## Discussion

All plans produced post-script implementation met all clinical goals and were considered clinically acceptable following the local plan checking and approval process. Introduction of the KBP script reduced the population-averaged mean rectum dose from 41.6Gy in the historic cohort to 36.0Gy in the post-KBP script implementation cohort (see Table 1).

A more robust method for comparison that considers variation in dose with PTV-rectum overlap is to examine the change in the curve in Fig. 2 following implementation of the KBP tool. A new MARD curve fitted to the post-KBP data was found to have *D*_*mean*_^*rectum*^/*D*_*Px*_ on average 0.05 lower, corresponding to a reduction in D_rec_ = 3.7Gy for D_Px_ = 74Gy. This change corresponds to the data being approximately equivalent to the OARD curve established pre-KBP implementation, suggesting that the post-implementation median level of rectal sparing has now converged on the previously optimal practice.

Similarly, individual cases in the historical cohort are positioned well above the original MARD curve implying that rectum dose was sub-optimal for these patients, but comparison of the curves describing the outlying plans before and after implementation of the script showed that *D*_*mean*_^*rectum*^/*D*_*Px*_ reduced by an average 0.07 corresponding to a change in D_rec_ = 5.2Gy for D_Px_ = 74Gy. This means that post-implementation the least optimal plans are now of similar quality to the median levels achieved prior to implementation, which might be expected as the planners were always asked to obtain the median expected value or better.

From Fig. 1 it can be seen that whilst the general trend reported by Moore et al. (i.e. increased overlap results in increased OAR dose) is observed in the historical cohort the local data is much shallower than the Moore et al. model. This suggests that historically local patients exhibiting a small overlap between rectum and PTV1 were being planned with doses only moderately lower than patients with much larger overlap volumes suggesting that the planner stopped optimising the plan too soon in these instances. Post script implementation (see Fig. 2) average rectum doses are reduced overall but for a few patients exhibiting lower overlap volumes (V_ovr_/V_rec_ < =0.2) the reduction in average rectum dose was more pronounced. If a new curve was drawn to represent the optimum average rectum dose post-script implementation the new curve would be much steeper and closer to that proposed by Moore et al. This indicates that introducing the script has increased the steepness of the local data by encouraging planners to continue optimising plans where further gains are most achievable. This represents a potentially significant gain in plan quality from this simple application however further data is required to confirm the steeper trend post-script implementation.

In order to assess the impact of this tool on clinical practice the data mining script was used to extract plan statistics from the post-KBP script implementation cohort which were compared to the same results from the historical cohort (see Table 1). The difference between D99 and D1 for PTV3, D99 for PTV2 and D99 for PTV1 before and after implementation of the script was less than 0.5Gy. This change is not statistically significant (applying a *t*-test with 0.05 significance level) implying that target coverage has been unaffected by the KBP script. The average plan MU increased post-script implementation from 427MU to 466MU indicating that the script instigated a statistically significant increase in plan complexity. This result was predicted as improving rectal sparing whilst maintaining other aspects of plan quality can only be achieved through greater plan modulation. Whilst statistically significant this increase in plan MU is modest and significantly below the upper threshold of 600MU applied as part of this study. The average mean bladder dose reduced slightly by 0.9Gy following script implementation. This small reduction in dose is not statistically significant but confirms that the script induced increase in rectal sparing has not generated an unforeseen increase in bladder dose. Average bowel dose has decreased by 3.8Gy post-script implementation. This decrease is not statistically significant but indicates that the script has introduced a desirable trend for lower bowel doses. This is likely a consequence of increased dose conformity at the posterior surface of the PTV structures created whilst attempting to spare the rectum. Whilst the dose to the aforementioned OAR structures has remained stable or reduced post-script implementation the dose to both femoral heads has increased. This is a direct result of the script encouraging planners to push dose from the rectum OAR resulting in lateral low dose spread towards the femoral heads. The increase in lateral dose spread is also observed as a statistically significant reduction in CI (see Table 1). Both the increase in femoral head dose and lateral dose spread were foreseen prior to script implementation and the risk accepted by local clinicians and controlled via plan quality constraints. Furthermore the average femoral head dose and CI values are well within accepted clinical tolerances and are therefore of low concern.

Whilst the change in femoral head dose is not of clinical concern, since it tends to fall easily well within clinical tolerance, it does raise a question about the nature of the plan improvements induced by the KBP tool. The pre-implementation study revealed no apparent change in femoral head dose compared to original clinical plan when re-planned for both the “sub-optimal” and “optimal” cohorts however the sub-optimal cohort demonstrated a benefit in rectum dose. This implies that the KBP metric was able to identify plans that were legitimately sub-optimal (i.e. not on the pareto optimal front). However, when actively employing the tool to guide planners an increase in femoral head dose is observed relative to the pre-KBP population. This perhaps indicates that, for some plans at least, the KBP tool has induced a shift along the pareto front rather than shifting a sub-optimal plan onto the pareto front. This may especially be true for those patients where the mean rectum dose is significantly below OARD. RayStation MCO module allows users to investigate the trade-offs whilst moving across the pareto front and should enable the production of pareto optimal plans [9, 10]. This approach is different to KBP planning in that it does not directly rely upon previous local knowledge to produce optimal plans. MCO planning, however, does require user specified trade-off optimisation functions and constraints in order to produce an initial collection of pareto plans. These plans are then examined and explored by the user in order to produce the final clinical plan. The quality of the final optimal plan is therefore heavily reliant upon the initial user-specified trade-off functions. It is therefore important that thorough commissioning work is performed to ascertain the most suitable initial set of trade-off functions for a given clinical site in in order to ensure that a truly pareto optimal final plan is produced. The MCO planning module has not been commissioned for clinical use locally however the KBP tool presented in this work could be used to assess whether plans generated by MCO are, in terms of average rectum dose, comparable to (or better than) those automated manually acting as a valuable aid during the commissioning process. The KBP tool presented here can therefore be used as an alternative to MCO or to compliment MCO to QA final plan quality.

The reduction in population-averaged mean rectum dose induced by implementation of the KBP script is primarily achieved via a reduction in the amount of moderate to low-dose (i.e. <= 50Gy) delivered to the rectum. This is illustrated in Table 1 where the average V30, V40 and V50 (expressed as a fraction of total rectum volume) are statistically significantly lower for the population of plans produced post-script implementation compared to the historical cohort. In contrast, the average V60, V65 and V70 are approximately the same for the post-script and historical cohorts. This result is to be expected as the volume of rectum receiving the highest doses will be the region overlapping the PTV and therefore achievable dose reduction to this region is more limited without compromising target coverage. One possible concern to driving the optimisation of a plan using the KBP tool is that we may increase dose elsewhere, particularly around the rectum. However, the dose within 1 cm of the rectum did not increase as a function of δ-values, suggesting that plans optimised using the KBP tool remain robust to intrafractional rectum changes.

Reports in the literature are mixed about the clinical benefits of reducing moderate-low dose exposure to the rectum. The QUANTEC review concluded that high dose limits (> = 60Gy) were of greater significance in terms of rectal toxicity than lower doses (<60Gy) [11]. This conclusion is supported by Fiorino et al. [12], Tucker et al. [13] and Michalski et al. [14] whose studies found correlations between grade 2 rectal toxicity and high dose volumes only. However there also exist a significant number of studies reporting that the extent of low/intermediate dose rectum exposure correlates with rectal toxicity. For example, Buettner et al. [15] investigated the shape of the dose distribution across the rectum and the correlation with toxicity reporting a correlation between rectal bleeding and doses between 40-60Gy. Gulliford et al. [16] reported a reduction in the incidence of moderate/severe rectal toxicity with dose reduction across the whole DVH concluding that lower doses are of clinical importance. Although investigating hypofractionated treatments, Kim et al. [17] reported a strong correlation between grade 2+ delayed rectal toxicity and the percentage of rectal wall circumference receiving low dose. There is therefore evidence to suggest that the reduction in low/moderate dose exposure to the rectum, driven by the introduction of the KBP script, has a real clinical benefit to the patient in terms of reduced risk of rectal toxicity. Follow-up work from this study will investigate the feasibility of making more limited gains by optimising the dose in the rectal-PTV overlap region.

## Conclusions

RayStation has been used successfully to guide planners on the expected value of a key plan quality metric, leading to significant reduction in population-averaged mean rectal dose, which may translate into reduced toxicity risk for patients. A shared knowledge base with other RayStation users would be desirable to allow centres to benchmark local plan quality against that achieved elsewhere and drive overall improvement.

## Abbreviations

CI: Conformity index
DVH: Dose volume histogram
HR: High risk
KBP: Knowledge based planning
LR: Low risk
MARD: Median average rectum dose
MCO: Multi Criteria Optimisation
OAR: Organ at risk
OARD: Optimum average rectum dose
OVH: Overlap volume histogram
PTV: Planning target volume
VMAT: Volumetric modulated arc therapy
WOC: Worcestershire Oncology Centre

## References

[1] Moore KL, Brame RS, Low DA, Mutic S (2011). Experience-based quality control of clinical intensity-modulated radiotherapy planning. Int J Radiat Oncol Biol Phys.

[2] Wu B, Ricchetti F, Sanguineti G, Kazhdan M, Simari P, Chuang M (2009). Patient geometry-driven information retrieval for IMRT treatment plan quality control. Med Phys.

[3] Appenzoller LM, Michalski JM, Thorstad WL, Mutic S, Moore KL (2012). Predicting dose-volume histograms for organs-at-risk in IMRT planning. Med Phys.

[4] Schreibmann E, Fox T (2014). Prior-knowledge treatment planning for volumetric arc therapy using feature-based database mining. J Appl Clin Med Phys.

[5] Nwankwo O, Mekdash H, Sihono DH, Wenz F, Glatting G (2015). Knowledge-based radiation therapy (KBRT) treatment planning versus planning by experts: validation of a KBRT algorithm for prostate cancer treatment planning. Radiat Oncol.

[6] Wang Y, Zolnay A, Incrocci L, Joosten H, McNutt T, Heijmen B (2013). A quality control model that uses PTV-rectal distances to predict the lowest achievable rectum dose, improves IMRT planning for patients with prostate cancer. Radiother Oncol.

[7] Good D, Lo J, Lee WR, Wu QJ, Yin FF, Das DK (2013). A Knowledge-Based Approach to Improving and Homogenizing Intensity Modulated Radiation Therapy Planning Quality Among Treatment Centers: An Example Application to Prostate Cancer Planning. Int J Radiat Oncol Biol Phys.

[8] Song T, Li N, Zarepisheh M, Li Y, Gautier Q, Zhou L, Mell L, Jiang S, Cervino L (2016). An Automated Treatment Plan Quality Control Tool for Intensity-Modulated Radiation Therapy Using a Voxel-Weighting Factor-Based Re-Optimization Algorithm. PLoS ONE.

[9] Kierkels R, Visser R, Hendrik B, Langendijk J, van’t Veld A, Steenbakkers R, Korevaar E (2015). Multicriteria optimization enables less experienced planners to efficiently produce high quality treatment plans in head and neck cancer radiotherapy. Radiat Oncol.

[10] McGarry C, Bokrantz R, O’Sullivan J, Hounsell A (2014). Advantages and limitation of navigation-based multicriteria optimisation (MCO) for localized prostate cancer IMRT planning. Med Dosim.

[11] Michalski JM, Gay H, Jackson A, Tucker SL, Deasy JO (2010). Radiation dose-volume effects in radiation-induced rectal injury. Int J Radiat Oncol Biol Phys.

[12] Fiorino C, Fellin G, Rancati T, Vavassori V, Bianchi C, Borca VC (2008). Clinical and dosimetric predictors of late rectal syndrome after 3D-CRT for localized prostate cancer: Preliminary results of a multicenter prospective study. Int J Radiat Oncol Biol Phys.

[13] Tucker S, Dong L, Michalski JM, Bosch WR, Winter K, Cox JD (2012). Do intermediate radiation doses contribute to late rectal toxicity? An analysis of data from RTOG 94–06. Int J Radiat Oncol Biol Phys.

[14] Michalski JM, Yan Y, Watkins-Bruner D, Bosch W, Winter K, Galvin JM (2013). Preliminary toxicity analysis of 3DCRT versus IMRT on the high dose arm of the RTOG 0126 Prostate Cancer Trial. Int J Radiat Oncol Biol Phys.

[15] Buettner F, Gulliford SL, Webb S, Sydes MR, Dearnaley DP, Partridge M (2009). Assessing correlations between the spatial distribution of the dose to the rectal wall and late rectal toxicity after prostate radiotherapy: an analysis of data from the MRC RT01 trial (ISRCTN 47772397). Phys Med Biol.

[16] Gulliford SL, Foo K, Morgan RC, Aird EG, Bidmead AM, Critchley H (2010). Dose-volume constraints to reduce rectal side effects from prostate radiotherapy: evidence from MRC RT01 trial ISRCTN 47772397. Int J Radiat Oncol Biol Phys.

[17] Kim DW, Cho LC, Straka C, Christie A, Lotan Y, Pistenmaa D (2014). Predictors of rectal tolerance observed in a dose-escalated phase 1–2 trial of stereotactic body radiation therapy for prostate cancer. Int J Radiat Oncol Biol Phys.

